# Understanding the privacy disclosure behavior and privacy concerns on short-form video platforms: an empirical investigation

**DOI:** 10.3389/fpsyg.2025.1535533

**Published:** 2025-09-23

**Authors:** Heng Lu, Aiye Wei, Xin Li

**Affiliations:** ^1^Department of Journalism and Communication, Shaanxi Normal University, Xi’an, China; ^2^Key Laboratory of Folk Song Intelligent Computing and Service Technology, Ministry of Culture and Tourism, Xi’an, China

**Keywords:** privacy disclosure behavior, short video, institutional privacy concerns, peer privacy concerns, influencing factors

## Abstract

In recent years, platform user privacy leaks have occurred frequently, heightening users’ privacy concerns, which may directly affect their behavior on short video platforms. Based on communication privacy management theory, this study constructs a conceptual model of factors influencing the privacy disclosure behavior of short video users from the perspectives of both peer privacy concerns and institutional privacy concerns. Data from 302 short video users were collected via an online questionnaire and analyzed using structural equation modeling. The results indicate that perceived peer risk and information sensitivity positively influence peer privacy concerns; the effectiveness of privacy protection technology and privacy policies negatively influence institutional privacy concerns; information sensitivity also positively affects institutional privacy concerns; both institutional and peer privacy concerns negatively affect users’ privacy disclosure behavior; and institutional privacy concerns positively influence peer privacy concerns. The study discusses how these findings extend the application of communication privacy management theory in the context of short video platforms, highlighting the intertwined nature of institutional and peer-induced privacy risks. Practical implications for platform designers and policymakers are offered to enhance user trust and promote responsible privacy management.

## 1 Introduction

Videos contain richer forms of personal information than text or static images, encompassing facial, physical, and voice features Kang et al. (2022). Consequently, users may disclose more personal information when engaging with short video platforms. The increasingly accurate and intelligent recommendation algorithms of these platforms may heighten users’ institutional privacy concerns. Furthermore, as the social features of short video platforms continue to evolve, privacy threats from peers may amplify users’ concerns, potentially influencing their privacy disclosure behaviors (Ozdemir et al., 2017). The content generated by users is a crucial factor for the survival and development of short video platforms. Users’ concerns regarding peer and institutional privacy may affect both their willingness to engage with the platform and their usage patterns, which in turn impacts the platforms’ growth. Therefore, it is of great theoretical and practical significance to explore the two facets of privacy concerns on the factors influencing the privacy leakage behavior of short video users.

The majority of studies begin with institutional privacy issues, pointing out that users’ security and privacy may be threatened by the information practices of websites, online platforms, and other organizations. Users become concerned about platforms and organizations violating their privacy as a result of this threat (Xu et al., 2011). Such institutional privacy concerns can directly affect users’ privacy disclosure behavior (Xu et al., 2013). A few studies have found that users’ privacy concerns not only originate from institutions but also exist in peer-oriented online interactions. This threat of privacy invasion from online peers is defined as peer privacy concerns (Zhang et al., 2022). The impact of a single privacy concern on users’ privacy disclosure behavior has been extensively studied in previous research, but it is unclear how the two privacy concern perspectives—which refers to the conflict between institutional and peer privacy—affects users’ privacy disclosure behavior on short video platforms and how the two facets of privacy concerns relate to one another. Furthermore, the impact of users’ privacy concerns on privacy disclosure behavior in short video platforms is not included in the current research on users’ privacy disclosure behavior, which focuses on items like social media SNS (Jozani et al., 2020), e-commerce platforms (Dinev and Hart, 2006), online medical platforms (Young et al., 2013), and financial websites (Xu et al., 2011). It is found that short video users face the two privacy concerns from institutions and peers. It is difficult to fully reveal the mechanism of privacy concerns in the privacy disclosure behavior of short video users from the perspective of institutional privacy concerns alone.

Based on the above reality and theoretical background, this paper proposes the following research questions: (1) How do privacy concerns affect users’ privacy disclosure behavior on short video platforms? (2) How do the two facets of privacy concerns work in tandem to impact user’s privacy disclosure behavior? (3) What are the antecedents of institutional and peer privacy concerns? In order to address the above questions, with communication privacy management (CPM) as the theoretical basis, this study examines the facets of concerns and benefits that may affect user’s privacy disclosure behavior on short video platforms.

This study makes two significant contributions to the IS literature. First, it demonstrates the necessity of incorporating the two privacy concerns in short video users’ privacy behavior research. Existing studies predominantly focus on single-dimensional institutional privacy concerns while neglecting the role of peer privacy concerns. This study innovatively introduces a dual privacy concerns framework, thoroughly elucidating the influencing mechanisms of users’ privacy disclosure behaviors based on the unique characteristics of short video platforms, thereby expanding the research perspective on short video users’ privacy behaviors. Second, it clarifies the formation mechanism of dual privacy concerns in the short video context. Current privacy concern research mainly concentrates on social media and e-commerce contexts, with limited exploration of the interrelationships between different privacy concerns. Grounded in CPM, this study examines how three boundary management factors (boundary rules, coordination, and turbulence) influence dual privacy concerns, while also verifying the moderating effect of institutional privacy concerns. These findings broaden the research paradigm of privacy concerns from the theoretical perspective of CPM.

## 2 Literature review

### 2.1 Privacy concerns

Privacy concerns reflect Internet users’ anxieties regarding how their personal information is collected and used by websites, highlighting perceived gaps between expected and actual data handling practices (Hong and Thong, 2013). Research primarily distinguishes two dimensions: institutional privacy concerns and peer privacy concerns. (1) Institutional Privacy Concerns: Stemming from user apprehensions about how platforms, governments, and other organizations manage personal data—specifically unauthorized access, secondary use, interception, and sale to third parties (Liu et al., 2022; Wang et al., 2019). Studies explore how these concerns influence user behaviors, including privacy disclosure (Xu et al., 2013), reduced social media engagement (Neves et al., 2022), and the privacy paradox (Livingstone, 2008). For instance, Dinev and Hart (2006) found they significantly impact disclosure willingness, while Xu et al. (2011) examined how perceptions of institutional protection shape these concerns. (2) Peer Privacy Concerns: Arise when users feel unable to maintain personal boundaries online due to peer behavior (Zhang et al., 2022). Zhang et al. conceptualized these across four dimensions: information, psychological, virtual territory, and communication privacy concerns. As social networks evolve, online privacy faces threats not only from institutions but increasingly from peers, heightening the prominence of peer privacy issues (Dinev et al., 2013). Research investigates both antecedents and consequences: Ozdemir et al. (2017) used the APCO framework to show how privacy experiences and awareness intensify peer concerns, reducing disclosure willingness on social media. Similarly, Nguyen et al. (2024) proposed a dual peer privacy calculus model linking these concerns to active and passive social networking site use.

While existing research has explored various factors influencing short video users’ privacy disclosure, it predominantly relies on privacy calculus theory to explain the privacy paradox. Studies on user privacy concerns have largely focused on institutional (platform-level) concerns, overlooking the impact of peer privacy concerns. Although some work suggests peer concerns significantly reduce disclosure on social media, these findings stem from traditional platforms and fail to address the unique privacy implications of visual content and algorithmic recommendations inherent to short video platforms. Furthermore, current research inadequately examines the interaction between these dual concerns—specifically, how institutional concerns may moderate the effect of peer risks on disclosure, a relationship needing careful analysis within short video contexts. To bridge these gaps, this study introduces a dual privacy concerns framework and analyzes short video users’ disclosure behaviors through CPM, focusing on boundary rules, turbulence, and coordination. This approach aims to uncover the mechanisms governing privacy concerns in short video disclosure contexts.

### 2.2 Users’ privacy disclosure behaviors

User privacy disclosure behavior refers to the behavior of users who voluntarily disclose personal information such as names, preferences, and demographics to Internet platforms (Wang et al., 2021). The conflict between the necessity of privacy disclosure and the risk of privacy leakage is a key factor affecting users’ use or continued use of Internet platforms (Hong and Thong, 2013). The rise of short video platforms has introduced novel privacy challenges. The visual nature of their content format (e.g., exposure of facial features and geolocation data) may amplify risks of sensitive information leakage (Kang et al., 2022), while algorithm-driven precision recommendation mechanisms further blur privacy boundaries.

Most of the related studies are empirical analyses of theoretical model construction based on the theories of privacy calculus theory (Dinev and Hart, 2006), the theory of planned behavior (Xu et al., 2013), social exchange theory (Liu et al., 2016), social exchange theory (Loiacono, 2015), uses and gratifications theory (Hollenbaugh and Ferris, 2014), and social influence process (Min and Kim, 2015). In addition, many scholars have explored the relationship between privacy concerns and privacy disclosure behavior. However, the conclusions of many studies are inconsistent. For example, Nemec Zlatolas et al. (2015) and Wang J. et al. (2024) found that privacy concerns are an important factor influencing users’ self-disclosure behaviors, while Krasnova et al. (2010) found that there is no significant relationship between privacy concerns and privacy disclosure behaviors in their study. Therefore, the relationship between privacy concerns and users’ privacy disclosure behavior still needs to be further explored. Several scholars have investigated the influencing factors of privacy disclosure behaviors among short video users. For instance, Zhu et al. (2022) examined how interpersonal and human-computer interactions affect users’ privacy disclosure intentions through perceived risks and perceived benefits from an interactive perspective. Li et al. (2023) analyzed the impact of user behavior and benefits obtained on privacy concerns and disclosure behavior through user data. Kang et al. (2022) explored the impact of parental intervention on adolescents’ privacy disclosure behaviors and risk perceptions in short video contexts.

Esmaeilzadeh (2024) and Neves et al. (2022) studied privacy disclosure in a dual privacy context. But they focused on health information and the social networking sites. How short video users disclose personal information under dual privacy concerns remains unclear. Furthermore, existing studies have inadequately addressed the interaction between these dual concerns, particularly how institutional privacy concerns may moderate the effect of peer risks on disclosure behaviors (Neves et al., 2022).

### 2.3 Communication privacy management theory

In 1991, Petronio proposed the Theory of Communication Boundary Management (CBM) and applied it to privacy management between couples (West and Turner, 2014). In 2002, Petronio further elaborated on this theory and formally renamed it the Theory of Communication Privacy Management (CPM). CPM is used to explain the negotiation process individuals undergo when disclosing information and determining whether access to that information poses potential risks (Petronio, 2002). The theory suggests that individuals maintain and coordinate privacy boundaries with various communicating parties by establishing these boundaries based on the delineation between personal and public information. Consequently, individuals form a region of information with well-defined boundaries, determining what information is private. Any attempt to breach these boundaries may be perceived as a threat to privacy, giving rise to privacy concerns (Xu et al., 2011). Meanwhile, when people want or need to disclose private information, they make judgments about to whom they decide to open their privacy boundaries in order to achieve their goal of revealing or concealing their private information (Smith and Brunner, 2017).

Communication Privacy Management theory conceptualizes privacy management through three interdependent elements: boundary rules, coordination, and turbulence (Xu et al., 2011). These elements capture the core processes and challenges individuals face in managing private information. Boundary rules govern when and how individuals disclose personal information (De Wolf et al., 2014). Boundary coordination occurs when private information is shared, involving negotiated rule-setting between parties (e.g., trading privacy control for compensation; Metzger, 2007). Boundary turbulence arises when rules are ambiguous or coordination fails, prompting behavioral adjustments (West and Turner, 2014). CPM is now widely applied to digital privacy contexts, including: Enterprise social networks (Wang Y. et al., 2024), Hotel social robots (Jia et al., 2024), Chatbots (Liu et al., 2024). For instance, Liu et al. (2022) used CPM to explore consumer vulnerability on shared lodging platforms, while Wang et al. (2019) developed a CPM-based model clarifying how privacy violations amplify concerns.

The process of users utilizing short videos constitutes a process of boundary management between users and platforms. In the context of short video platforms, boundary rules refer to users’ self-established conditions for information disclosure (e.g., “not sharing geolocation data”); boundary coordination involves negotiating rule implementation with either the platform or peers (e.g., accepting privacy policies in exchange for service access); and boundary turbulence triggers remediation mechanisms when rules are violated (e.g., adjusting permission settings). Short video users employ the CPM to implement dual privacy boundary management. Regarding peer privacy boundaries, users dynamically adjust their sharing strategies based on feedback from fan interactions. For institutional privacy boundaries, users determine their information disclosure levels according to the transparency of platform privacy policies and the trustworthiness of technological safeguards. Platforms engage in boundary coordination with users through privacy agreements, while technical vulnerabilities or ambiguous policies may trigger boundary turbulence (Xu et al., 2011; Petronio, 2002).

## 3 Constructs and hypotheses in the model

**Figure 1:**
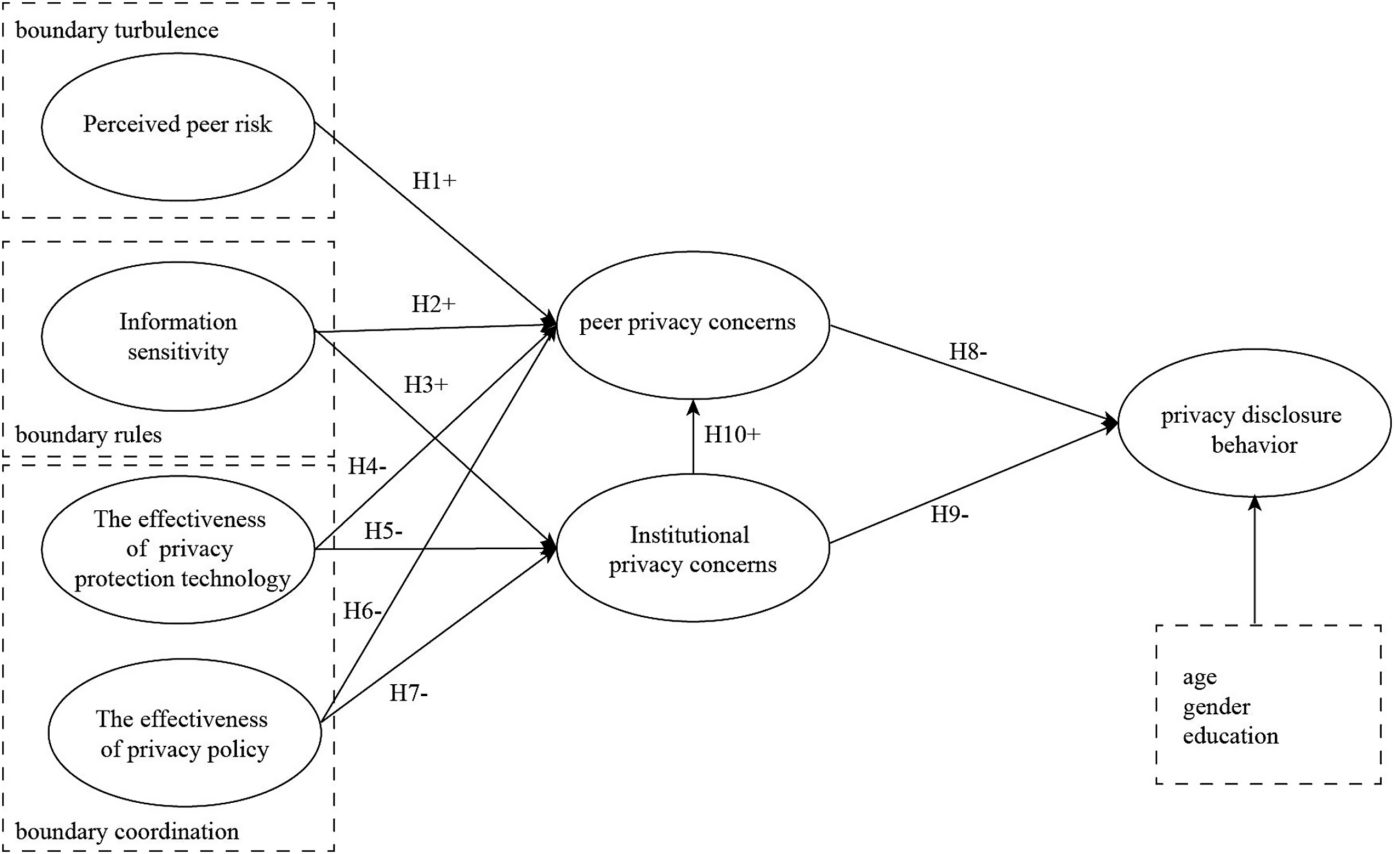
Research model.

This paper analyzes the factors influencing the privacy disclosure behavior of short video users from the perspective of institutional privacy concerns and peer privacy concerns, based on CPM. This study investigates the current status of short video users’ usage behavior, refines the factors affecting their privacy concerns, and combines the theoretical foundation of this paper to propose the antecedent variables of their institutional privacy concerns and peer privacy concerns. It constructs a model of the factors affecting short video users’ privacy disclosure behavior, as shown in Figure 1.

### 3.1 Perceived peer risk

Perceived peer risk refers to an individual’s subjective assessment of the potential privacy violations by online peers (Ozdemir et al., 2017). Grounded in Communication Privacy Management (CPM) theory, unauthorized information sharing constitutes a significant privacy threat (Petronio, 2002). Empirical studies demonstrate that while individuals exhibit higher trust toward familiar groups (Foddy et al., 2009), such trust diminishes when peer networks expand and risk perception emerges, subsequently triggering defensive behaviors. Although short-video platforms provide privacy settings for information access control, user data remain vulnerable to secondary dissemination by peers (Nemec Zlatolas et al., 2019). The platforms’ recommendation algorithms, which synthesize multi-dimensional data including contact lists, social connections, and geolocation information, often lead to private information leakage into public domains. When user-disclosed content is redistributed or utilized by online peers without consent, it violates users’ predetermined privacy boundary rules, thereby inducing privacy boundary turbulence. Therefore:

*H1*: Perceived peer risk positively affects users' peer privacy concerns.

### 3.2 Information sensitivity

Information sensitivity refers to the degree of psychological discomfort individuals experience when disclosing specific types of information (Dinev et al., 2013), constituting a fundamental determinant in establishing privacy boundary rules (Malhotra et al., 2004). Grounded in CPM, users construct virtual boundaries to regulate access to personal information, wherein information sensitivity governs their disclosure scope to both platforms and peers (Kolotylo-Kulkarni et al., 2021). Empirical studies consistently demonstrate users’ propensity to avoid disclosing sensitive information, with platforms’ requests for highly sensitive data frequently resulting in user opt-out behaviors (Malhotra et al., 2004). When handling highly sensitive information, short-video users exhibit heightened vigilance toward potential rights infringements stemming from privacy breaches (Yang et al., 2008). This heightened risk perception engenders privacy concerns through two distinct mechanisms: (1) peer privacy concerns emerge through social risk diffusion processes inherent in short-video platforms’ design. The platforms’ social visibility features create vulnerability to secondary data exposure, where sensitive information may be unexpectedly disseminated by peers through resharing or screenshotting, thereby inducing relational anxiety and boundary turbulence. (2) Institutional privacy concerns develop through technological trust erosion when platforms request information exceeding users’ sensitivity thresholds or fail to provide adequate transparency regarding data usage. Such practices foster perceptions of control asymmetry and trust deficit, leading users to question both the legitimacy of platforms’ data processing frameworks and the ethical boundaries of algorithmic data extraction and commercial exploitation (Maseeh et al., 2023). These dual pathways ultimately shape users’ comprehensive assessment of privacy threats in the short-video ecosystem. Therefore:

*H2*: Information sensitivity positively affects users' peer privacy concerns.

*H3*: Information sensitivity positively affects users' institutional privacy concerns.

### 3.3 The effectiveness of privacy protection technology

Privacy protection technology effectiveness reflects users’ perceived ability of platform safeguards to secure personal information (Wang et al., 2019). Within Communication Privacy Management (CPM) theory, these technologies serve as boundary management tools. Research confirms they significantly reduce inappropriate data access (Popp and Poindexter, 2006), while evolving standards enable personalized privacy control in mobile contexts (Duan and Wang, 2015). Although most major short-video platforms hold ISO27001 and ISO27701 certifications—indicating mature safeguards—breaches still occur through hacker attacks, eroding user trust. When protection efficacy appears insufficient, users’ concerns about institutional data misuse amplify (Maseeh et al., 2023), intensifying institutional privacy concerns.

Furthermore, effective privacy protection technologies affect not only the user-platform boundary but also privacy management between users and peers. Platform safeguards—like access controls, data encryption, screenshot blocking, and restricted resharing tools—directly limit peers’ ability to access or distribute sensitive user data without authorization. When users perceive robust technical measures preventing peer misuse (e.g., unauthorized forwarding, downloading, or screenshots), their concerns about peer boundary violations and resulting turbulence (i.e., peer privacy concerns) decrease significantly. Ultimately, technological efficacy strengthens users’ perceived control over peer information flows, preventing perceived risks from escalating into substantive peer privacy concerns. Therefore:

*H4*: The effectiveness of privacy protection technology negatively affects peer privacy concerns.

*H5*: The effectiveness of privacy protection technology negatively affects institutional privacy concerns.

### 3.4 The effectiveness of privacy policy

The effectiveness of privacy policy refers to the extent to which users perceive that the published privacy policy actually provides accurate and reliable information about the operator’s information practices (Xu et al., 2011). It is a process of boundary coordination that occurs through the collective control of users’ personal information, furthermore, credible and comprehensive privacy policies help platforms build a positive image among users. In addition, providing a detailed privacy policy is positively associated with users’ trust in the operator (Wu et al., 2012), which mitigates the perceived privacy risk. When users register and log in, links to privacy protection policies—often referred to as “Privacy Policy” and “User Service Agreement”—appear. These links serve to inform users about their rights and how their personal information will be gathered, used, stored, and shared while using the platform. Through the “Privacy Settings” feature, users of short videos can limit the amount of information that is stored and disclosed; however, platforms may obtain unauthorized access to users’ information in order to increase profits, which also puts users’ security and privacy at risk. Users’ institutional privacy concerns may rise when they believe short video services’ privacy policies are less effective.

Clear, transparent, and enforceable privacy policies—particularly those explicitly regulating how user data (especially user-generated content) may be utilized, shared, and disseminated among peers—play a pivotal role in managing privacy boundaries between users. For instance, policies that expressly prohibit unauthorized secondary dissemination (e.g., resharing, downloading, screenshot distribution) without users’ explicit consent, or that clearly delineate users’ control rights over content circulation (such as establishing the legal basis for “followers-only visibility” or “download prohibition” settings alongside platform enforcement commitments), can significantly reduce users’ anticipated risks of peer information misuse. When users trust that platforms will effectively constrain peer behaviors through policy enforcement measures (e.g., penalizing violative accounts, providing accessible infringement reporting mechanisms)—thereby upholding their established privacy boundary rules—their concerns about potential peer-induced boundary turbulence (i.e., peer privacy concerns) are correspondingly mitigated.

*H6*: The effectiveness of privacy policy negatively affects peer privacy concerns.

*H7*: The effectiveness of privacy policy negatively affects institutional privacy concerns.

### 3.5 Peer privacy concerns

Peer privacy concerns stem from worries about potential misuse of personal data shared with online peers, as highlighted by Raynes-Goldie (2010). Specifically, peers might disseminate private communications-including images, texts, emotions, and insights-meant for a restricted audience on social media. Similar to other privacy worries, intensified concerns over peers infringing on one’s privacy boundaries lead users to adopt precautionary measures, like refraining from using social networks or minimizing their exposure (Ozdemir et al., 2017). Consequently, users worried about peers violating their privacy tend to adjust their interactions, seeking to balance communication while reducing privacy risks to tolerable levels. Users with peer privacy concerns actively manage their privacy disclosures, often by limiting peer interactions, to maintain an acceptable level of privacy while using internet platforms (Neves et al., 2022). When users share short videos, this information reaches their peers, posing a potentially greater threat to user privacy (Ozdemir et al., 2017). Online peers could recognize individuals and objects in short videos, pinpoint users’ locations and activities, or reuse posted content without permission. Therefore:

H8: Peer privacy concerns negatively affect users’ privacy disclosure behavior.

### 3.6 Institutional privacy concerns

Another significant factor that motivates users to lessen their privacy disclosure behavior is institutional privacy concerns, which is defined as the degree of users’ anxiety regarding the exploitation of their personal information by platforms (Neves et al., 2022). According to CPM, users establish institutional trust boundaries through boundary rules when using short video platforms, explicitly defining expectations regarding “what information can be collected” and “how such information may be utilized.” When users perceive institutional behaviors transgressing their established privacy boundaries, boundary turbulence is triggered. To mitigate boundary violation risks, users strategically reduce privacy disclosures to achieve equilibrium, thereby diminishing opportunities for institutional information misuse (Zhang et al., 2022). This equilibrium enables continued platform engagement while allowing users to control or reduce their risk exposure (Neves et al., 2022). Existing research has demonstrated that institutional data collection processes may elicit individual privacy concerns (Maseeh et al., 2023). Therefore:

*H9*: Institutional privacy concerns negatively affect user privacy disclosure behavior.

Institutional privacy concerns not only directly influence disclosure behaviors but may also indirectly amplify users’ assessment of peer privacy risks by eroding their trust in the platform’s overall privacy protection capabilities. CPM posits that privacy boundary management constitutes a dynamic system where institutional and peer privacy boundaries exhibit synergistic effects (Petronio, 2002). When users experience heightened institutional privacy concerns, they may perceive the platform as incompetent in constraining peer behaviors (e.g., preventing unauthorized resharing or screenshot misuse). This systemic trust deficit lowers users’ tolerance threshold for peer privacy risks—even when the actual perceived peer risk remains unchanged, their pessimistic expectations about platform safeguards intensify peer privacy concerns.

*H10*: Institutional privacy concerns positively affect peer privacy concerns.

## 4 Methods

### 4.1 Resource identification initiative

TikTok is selected as the platform on which to test our model because it is one of the most widely used short video platforms in China. Up until March of 2021, the user market shares for TikTok was 59.88% which far surpassed those of short video applications. We developed an English questionnaire from the literature and then translated it into Chinese. Before distributing the questionnaire on a large scale, 10 heavy users of TikTok were first interviewed. Interviews yielded core findings. Users expressed worries that information shared with peers might be misinterpreted or misused (e.g., personal preferences taken out of context), which helped refine PPC measurements to fit short video scenarios. They distinguished between highly sensitive data (e.g., bank information) and low sensitivity data (e.g., username), aiding adjustments to IS items based on existing scales. Mentions of encryption tools’ practical impact on daily use guided wording tweaks for PPT items. The questionnaire items, originally referenced from established scales, were revised via these interviews to better align with the research context. After modification, 30 valid questionnaires were distributed on a small scale and recovered. Pre-test results showed more details. Items for PP needed specific adjustments (e.g., adding references to short video platform features). Responses clarified that PPR should focus on peers’ improper information handling, prompting wording refinements. Ambiguity in distinguishing platform vs. regulatory concerns in IPC items (based on prior scales) led to context-specific clarifications. Finally, the questionnaire items were modified and improved again according to the pre-test results. The measurement scale was finally generated (Table 1). All these constructs were measured using 1–5 Likert scale (1 = “strongly disagree,” 5 = “strongly agree”).

**Table 1:** Measurement instrument.

**Construct**	**Items**	**Sources**
Perceived peer risk (PPR)	PPR1: I think the information I share through TikTok could be used by someone to spy on me	Ozdemir et al. (2017)
	PPR2: I think the information I share through TikTok could be used against me by someone	
	PPR3: I think the information I share through TikTok could be used by someone to embarrass me	
	PPR4: I think the information I share through TikTok could be shared by someone with someone you do not want (e.g., “ex,” parents, teachers)	
Information sensitivity (IS)	IS1: I think TikTok collects too much personal information about me	Jozani et al. (2020)
	IS2: I think the information I provide to TikTok is very sensitive to me (e.g., ID number, home address, bank information, etc.)	
	IS3: I think I do not feel comfortable with the type of information TikTok request from me (e.g., requests for access to address book, requests for access to photo albums, etc.)	
The effectiveness of privacy protection technology (PPT)	PPT1: I believe TikTok is equipped with privacy protection technology	Wang et al. (2019)
	PPT2: I believe TikTok has reliable privacy protection technology	
	PPT3: I believe TikTok is secure in privacy protection technology	
	PPT4: I believe the third party is hardly getting my personal information from TikTok	
The effectiveness of privacy policy (PP)	PP1: I feel confident that TikTok’ privacy statements reflect their commitments to protect my personal information	Xu et al. (2011)
	PP2: With their privacy statements, I believe that my personal information will be kept private and confidential by TikTok	
	PP3: I believe that TikTok’ privacy statements are an effective way to demonstrate their commitment to privacy	
Peer privacy concerns (PPC)	PPC1: I am concerned that the information I share through TikTok with people I know could be misused by them	Neves et al., (2022)
	PPC2: I am concerned about sharing information through TikTok with people I know, because of what they might do with it	
	PPC3: I am concerned about sharing information through TikTok with people I know because they could use it in a way I did not foresee	
	PPC4: I am concerned that when I share information through TikTok with people that I know, those people may share it with others whom I did not intend	
	PPC5: I am concerned that the information I share through TikTok with people I know could be misinterpreted by them	
Institutional privacy concerns (IPC)	IPC1: I am concerned that the information I share through TikTok could be misused by TikTok and 3rd party affiliates	Neves et al., (2022)
	IPC2: I am concerned about sharing information through TikTok, because of what TikTok and 3rd party affiliates might do with it	
	IPC3: I am concerned about sharing information through TikTok, because TikTok and 3rd party affiliates could use it in a way I did not foresee	
	IPC4: I am concerned that when I share information through TikTok, they and 3rd party affiliates may share it with others whom I do not intend	
	IPC5: I am concerned that the information I share through TikTok could be misinterpreted by TikTok and 3rd party affiliates	
Privacy disclosure behavior(PDB)	PDB1: I keep sharing updates from my life on TikTok	Ozdemir et al. (2017)
	PDB2: I keep sharing what I want to express on TikTok	
	PDB3: I provide extra contact information (e.g., WeChat, email, or other social platform accounts) on TikTok to help others find me or attract more attention to me	
	PDB4: I keep my information up to date for my fans	
	PDB5: I will share a lot about myself on TikTok	
	PDB6: My TikTok give an idea of my preferences in music, movies, books, etc.	
	PDB7: My TikTok give an idea of who I am or what kind of person I am	

### 4.2 Sample and data collection

Our data were collected through wjx.cn, an online questionnaire collection platform. At the beginning of the questionnaire, we set a screening question asking the respondents whether they had any experience of using TikTok as users. Only those who chose “yes” could continue filling out the questionnaire. The sampling method used was convenience sampling. We targeted individuals who had prior experience with TikTok. This approach allowed us to efficiently gather responses from a relevant sample.

A total of 360 questionnaires were collected in this study, and after excluding invalid questionnaires, 302 valid questionnaires were finally collected. The statistical results show that the proportion of men and women is relatively balanced, and the proportion of female respondents is slightly higher than that of men; the age of 18–45 years old, the number of samples with education in the specialty and below is larger, which is basically in line with the status quo of the 20–39 years old group and the group of college-educated with the highest rate of short video usage, so the survey samples have a high degree of representativeness, and the results of the descriptive statistics of the surveyed respondents are shown in Table 2.

**Table 2:** Demographic statistics.

Attributes	Distribution	Number	Percentage
Gender	Male	148	49.01%
	Female	154	50.99%
Age	<18	34	11.26%
	18–25	78	25.83%
	26–35	67	22.19%
	36–45	53	17.55%
	46–55	45	14.90%
	>55	25	8.28%
Education	Junior college or below	166	54.97%
	Undergraduate	104	34.44%
	Postgraduate or above	32	10.60%

## 5 Analysis and results

In this study, SmartPLS3.0 was used for structural equation modeling and to validate the relationships between these variables. The reasons for choosing the PLS-SEM method to analyze sample data are as follows: (1) PLS is suitable for testing models with many constructs and complex relationships, and it can avoid infeasible solutions and factor uncertainty. (2) PLS has relatively low requirements for the sample size. In this study, the sample size is 302, which is more than 10 times the total number of measurement items (Hair et al., 2012). Additionally, control variables such as gender, age, and education level were incorporated into the PLS-SEM analysis. This addresses potential moderating effects. The impact is on relationships between PPC, IPC, and PDB. These control variables help eliminate potential confounding biases in the observed relationships.

### 5.1 Evaluation of measurement model

We analyse construct reliability and discriminant validity, by assessing the lowest indicator loading, the crossing loading, the average variance extracted (AVE), the heterotrait-monotrait ratio (HTMT), and the Fornell–Larcker criterion. Table 3 presents descriptive statistics. It also reports results of the Fornell–Larcker criterion. Table 4 shows discriminant validity. This is based on the cross-loading criterion. Table 5 provides values for Cronbach’s Alpha. It includes the lowest indicator loading. It also reports AVE and CR. Table 6 reports discriminant validity based on the HTMT criterion. To summarise, all Cronbach’s Alpha values and CR for all constructs are higher than 0.7, ensuring indicator reliability and convergent validity. All the AVE are higher than 0.5. All HTMT criterion scores but the relationship between PPC and IPC are below the threshold of 0.9, and the square root of the AVE of all constructs is higher than any correlation with another construct (Fornell-Larker criterion), indicating the presence of discriminant validity.

**Table 3:** Descriptive statistics and Fornell–Larcker criterion for model.

Construct	Mean	SD	PPR	IS	PPT	PP	PPC	IPC	PDB
PPR	3.906	0.886	**0.881**						
IS	4.217	0.782	0.491	**0.875**					
PPT	2.253	0.93	−0.406	−0.393	**0.758**				
PP	1.862	0.899	−0.384	−0.473	0.421	**0.843**			
PPC	4.241	0.787	0.537	0.741	−0.422	−0.502	**0.862**		
IPC	4.148	0.755	0.469	0.726	−0.509	−0.547	0.831	**0.876**	
PDB	1.882	0.800	−0.484	−0.616	0.48	0.480	−0.716	−0.711	**0.780**

Bold values on the diagonal are the square root of AVE values.

**Table 4:** Cross loadings, VIF, and method loadings for model.

Construct	PPR	IS	PPT	PP	PPC	IPC	PDB	Common loading
PPR1	**0.864**	0.418	−0.317	−0.28	0.447	0.396	−0.423	0.066
PPR2	**0.897**	0.452	−0.37	−0.381	0.527	0.438	−0.473	−0.055
PPR3	**0.892**	0.423	−0.364	−0.33	0.444	0.381	−0.400	0.014
PPR4	**0.873**	0.435	−0.379	−0.356	0.468	0.435	−0.405	−0.025
IS1	0.418	**0.852**	−0.29	−0.406	0.613	0.605	−0.545	0.02
IS2	0.444	**0.898**	−0.366	−0.422	0.682	0.662	−0.543	0.003
IS3	0.426	**0.874**	−0.372	−0.412	0.647	0.637	−0.529	0.34
PPT1	−0.195	0.027	**0.461**	0.068	0.018	−0.075	0.08	0.07
PPT2	−0.39	−0.209	**0.773**	0.252	−0.246	−0.336	0.325	−0.159
PPT3	−0.347	−0.356	**0.882**	0.386	−0.408	−0.477	0.459	−0.163
PPT4	−0.302	−0.404	**0.844**	0.402	−0.39	−0.452	0.41	0.097
EPP1	−0.284	−0.326	0.263	**0.76**	−0.350	−0.378	0.285	−0.073
EPP2	−0.343	−0.427	0.402	**0.897**	−0.476	−0.530	0.485	−0.01
EPP3	−0.341	−0.434	0.385	**0.866**	−0.430	−0.459	0.418	−0.039
PPC1	0.470	0.601	−0.359	−0.401	**0.864**	0.698	−0.641	0.037
PPC2	0.479	0.655	−0.361	−0.401	**0.875**	0.730	−0.654	−0.092
PPC3	0.449	0.617	−0.339	−0.427	**0.838**	0.685	−0.559	0.033
PPC4	0.468	0.671	−0.386	−0.452	**0.872**	0.731	−0.615	0.056
PPC5	0.451	0.647	−0.373	−0.483	**0.863**	0.738	−0.612	−0.012
IPC1	0.392	0.638	−0.443	−0.485	0.722	**0.886**	−0.65	0.112
IPC2	0.437	0.669	−0.484	−0.524	0.746	**0.887**	−0.639	−0.038
IPC3	0.392	0.632	−0.445	−0.453	0.720	**0.864**	−0.598	0.064
IPC4	0.439	0.637	−0.412	−0.502	0.757	**0.879**	−0.634	−0.131
IPC5	0.392	0.601	−0.444	−0.427	0.692	**0.863**	−0.591	−0.008
PDB1	−0.398	−0.502	0.393	0.385	−0.586	−0.602	**0.83**	−0.048
PDB2	−0.429	−0.492	0.476	0.452	−0.598	−0.614	**0.848**	−0.333
PDB3	−0.444	−0.551	0.374	0.398	−0.656	−0.611	**0.759**	−0.124
PDB4	−0.322	−0.462	0.311	0.364	−0.493	−0.463	**0.648**	0.166
PDB5	−0.388	−0.465	0.328	0.321	−0.513	−0.478	**0.784**	0.075
PDB6	−0.365	−0.470	0.359	0.355	−0.551	−0.564	**0.799**	0.243
PDB7	−0.268	−0.400	0.358	0.325	−0.474	−0.515	**0.775**	−0.026

Bold values indicate standardized outer loadings on the corresponding latent construct.

**Table 5:** Cronbach’s Alpha, lowest indicator loading, AVE, CR.

Quality criterion	PPR	IS	PPT	PP	PPC	IPC	PDB
Cronbach’s Alpha	0.904	0.846	0.779	0.796	0.914	0.924	0.891
Lowest indicator loading	0.864	0.852	0.461	0.759	0.838	0.863	0.648
AVE	0.777	0.765	0.575	0.711	0.743	0.767	0.608
CR	0.933	0.907	0.837	0.880	0.935	0.943	0.915

**Table 6:** Discriminant validity based on HTMT criterion for model.

Construct	PPR	IS	PPT	PP	PPC	IPC	PDB
PPR							
IS	0.56						
PPT	0.473	0.395					
EPP	0.449	0.572	0.448				
PPC	0.588	0.841	0.493	0.583			
IPC	0.511	0.82	0.593	0.629	0.904^a^		
PDB	0.532	0.707	0.567	0.555	0.787	0.778	

a The normal threshold of 0.9 is not met. However, IPC and PPC constructs have strong conceptual similarity, and their measures are likely to be strongly correlated. They used almost the same measurement questions, but with different subjects. However, considering other discriminant validity criteria are met, we are confident in the overall discriminant validity of our study constructs.

### 5.2 Common method bias

To address potential common method bias, multiple strategies were employed in this study. First, at the beginning of the questionnaire, respondents were explicitly informed that all responses would be kept anonymous and confidential, with clear instructions encouraging honest feedback to minimize response bias. Second, Harman’s single-factor test was conducted through exploratory factor analysis; the results showed that the first unrotated factor explained 44.77% of the total variance, which is below the 50% threshold (Podsakoff and Organ, 1986). Third, a common latent factor was incorporated into the measurement model. All items were converted into single-item constructs, and the main constructs were treated as second-order constructs. The results revealed that most loadings of the method factor were either non-significant, small, or negative (Table 4). Therefore, common method bias did not systematically interfere with the measurements.

### 5.3 Evaluation of structural model

**Figure 2:**
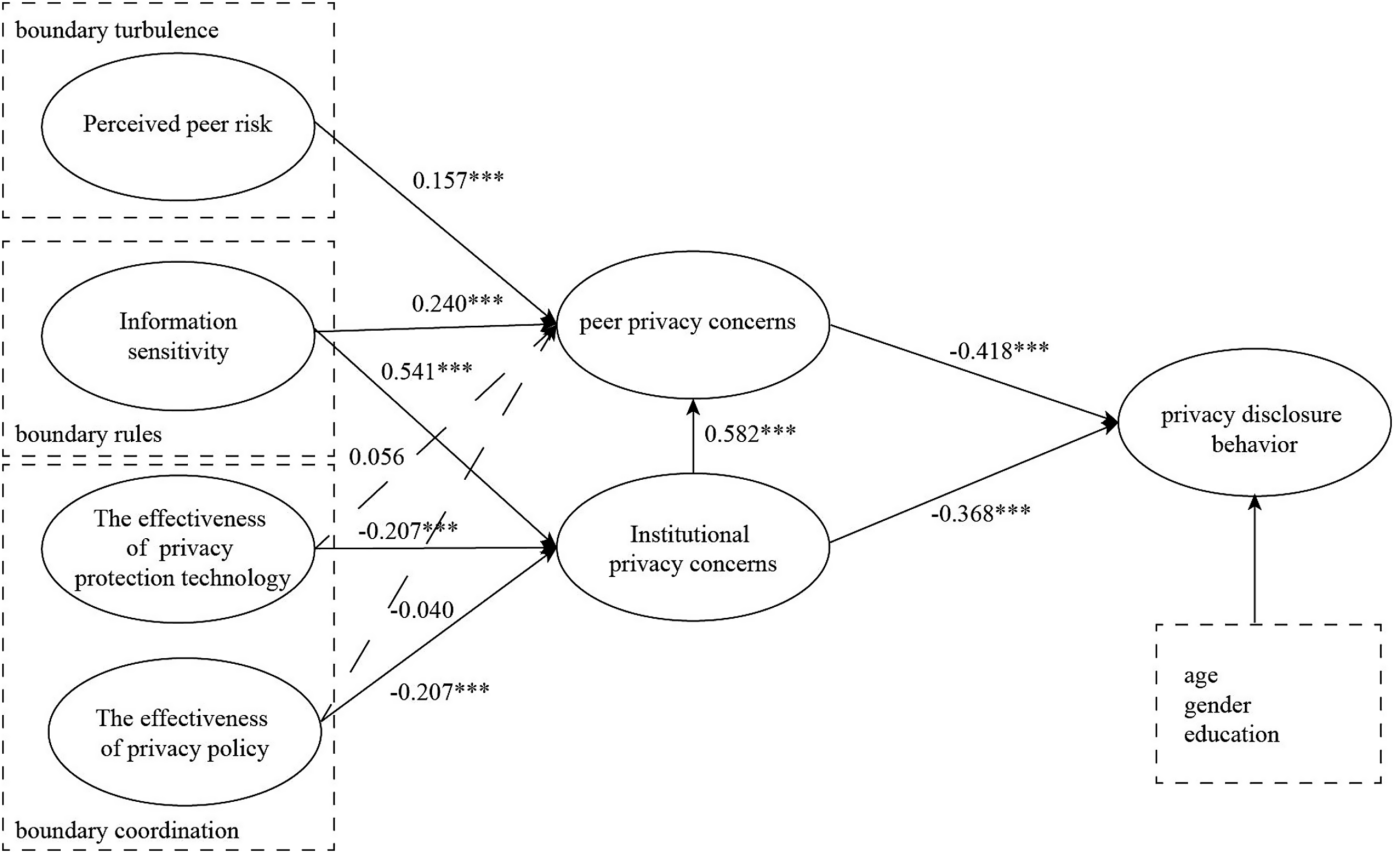
Model results. ***p < 0.001, **p < 0.01, *p < 0.05.

The SRMR for model is 0.06, which is below the threshold of 0.08. The NFI is 0.831. Although it is slightly below the 0.9 threshold, it is close to this value. This suggests that the model exhibits good fit. The model passed the most of hypothesis tests, see Figure 2 and Table 7. Peer privacy concerns and institutional privacy concerns have a significant negative effect on users’ privacy disclosure behavior. The effects of variables affecting institutional privacy concerns are as follows: information sensitivity has a significant positive effect, the effectiveness of privacy protection technology and the effectiveness of privacy protection policies has a significant negative effect. The effects of variables affecting peer privacy concerns are as follows: perceived peer risk has a significant positive effect, information sensitivity has a significant positive effect. In addition, institutional privacy concerns positively influence peer privacy concerns.

**Table 7:** Summary of hypothesis test results.

Hypothesis	Path coef	*p*-value	2.5%	97.5%	Decision
H1: PPR ➔ PPC	0.157***	<0.001	0.070	0.254	Supported
H2: IS ➔ PPC	0.240***	<0.001	0.120	0.364	Supported
H3: IS ➔ IPC	0.541***	<0.001	0.408	0.651	Supported
H4: PPT ➔ PPC	0.056	0.120	−0.016	0.126	Rejected
H5: PPT ➔ IPC	−0.207***	<0.001	−0.313	−0.116	Supported
H6: PP ➔ PPC	−0.040	0.355	−0.125	0.045	Rejected
H7: PP ➔ IPC	−0.207***	<0.001	−0.321	−0.098	Supported
H8: PPC ➔ PDB	−0.418***	<0.001	−0.630	−0.193	Supported
H9: IPC ➔ PDB	−0.368***	<0.001	−0.578	−0.155	Supported
H10: IPC ➔ PPC	0.582***	<0.001	0.439	0.708	Supported

***p < 0.001, **p < 0.01, *p < 0.05.

Table 8 presents the results of total indirect effects and specific indirect effects. The total indirect effect analysis shows that PPR and IS have significant negative total indirect effects on PDB. PPT and PP have significant positive total indirect effects on PDB. For specific indirect effects, PPR has a significant negative indirect effect on PDB through PPC. IS has significant negative indirect effects on PDB through both PPC and IPC. PPT and PP each have significant positive indirect effects on PDB through IPC.

**Table 8:** Summary of mediation analysis test results.

Indicators	Hypothesis	Path coef	*p*-value	2.5%	97.5%
Total indirect effect	PPR ➔ PDB	−0.066*	0.014	−0.126	−0.021
	IS ➔ PDB	−0.43***	<0.001	−0.529	−0.315
	PPT ➔ PDB	0.103**	0.009	0.032	0.188
	PP ➔ PDB	0.143***	<0.001	0.066	0.228
Specific indirect effect	PPR ➔ PPC ➔ PDB	−0.066*	0.014	−0.126	−0.021
	IS ➔ PPC ➔ PDB	−0.1**	0.007	−0.178	−0.036
	IS ➔ IPC ➔ PDB	−0.199***	<0.001	−0.326	−0.08
	PPT ➔ IPC ➔ PDB	0.076*	0.010	0.027	0.144
	PP ➔ IPC ➔ PDB	0.076*	0.021	0.023	0.15

***p < 0.001, **p < 0.01, *p < 0.05.

Among the control variables, gender was found to exert a significant negative effect on PPC (*β* = −0.079, *p* = 0.016). In contrast, gender, age, and education level showed no significant impacts on IPC and PDB. Additionally, age and education level had no significant influence on PPC.

## 6 Discussion

### 6.1 Perceived peer risk positively influences peer privacy concerns

Perceived peer risk positively and significantly affects peer privacy concerns and indirectly affects users’ privacy disclosure behavior through peer privacy concerns. This finding is consistent with the findings of Ozdemir et al. (2017) and Quan-Haase and Ho (2020). Higher peer risk increases the level of concern about peer threats, and the user’s perceived privacy risk increases further, which in turn leads to higher peer privacy concerns and ultimately affects their privacy disclosure behavior. It has also been shown that although most students are aware of the possible consequences of providing personally identifiable information on Facebook, they are not concerned about it (Govani and Pashley, 2007), which is inconsistent with the findings of this paper. Peer privacy concerns are correspondingly lower when the perceived benefits of online peers outweigh the perceived risks, such benefits may be self-presentation fulfillment, the need to maintain realistic social relationships, and higher online status.

Users exhibit varying levels of concern regarding different types of online peer privacy threats. In the context of short videos, users can categorize peers based on perceived risk, which in turn influences their decisions on content visibility. Selective posting according to peer group classification narrows the scope of information dissemination in short videos, fostering the formation of tighter, interest-based communities. Short videos may be followed by peers in posting and dissemination to form groups or communities centered on blood, geography, or interest, etc. Grouping peers can allow information to be disseminated in a smaller, more reliable circle. Platforms like WeChat allow users to specify who can see their posts, ranging from everyone to no one, thus enabling users to manage their privacy more effectively. When posting a short video, users can select options such as “Publicly visible,” “Not to be seen by anyone,” “Visible to friends,” “Partially visible,” and “Visible only to myself” to restrict the access rights of their peers. Short video platforms should enhance their grouping functionalities to allow for more granular control over content visibility based on factors such as geography, age, and gender. While users delegate some privacy rights to platforms, it is imperative for these platforms to safeguard user privacy by limiting the potential for data misuse and ensuring responsible handling of personal information.

### 6.2 Information sensitivity positively affects peer privacy concerns, institutional privacy concerns

Information sensitivity positively and significantly affects peer privacy concerns and institutional privacy concerns and indirectly affects users’ privacy disclosure behavior through peer privacy concerns and institutional privacy concerns. This finding is consistent with the findings of Malhotra et al. (2004) and Xu et al. (2013) that information sensitivity affects privacy concerns and that users will be more reluctant to disclose sensitive information compared to less sensitive information. When users perceive the requested information as sensitive, their perceived risk increases, which in turn affects privacy concerns and privacy disclosure behavior.

In general, the more personal information involved and the more sensitive it is, the higher the user’s concern about its privacy leakage, and the higher the user’s institutional privacy concern and peer privacy concern. From the user’s perspective, the personal information involved in short video platforms, such as identity card number, bank card information, home address, work information, etc., are all sensitive information, of which identity card and bank card information are the most sensitive to the user, and many users will not take the initiative to carry out real-name authentication when using short videos, and they will fill in the form only when they have no other choice, such as purchasing commodities in live broadcasting rooms or receiving red packets for cash withdrawal, and will fill in the Personal identification information. Conversely, many users tend to overlook the sensitivity of requests for access to device storage, address books, or geographical locations, often consenting without hesitation. In 2015, the U.S. government proposed the Consumer Privacy Bill of Rights, defining sensitive consumer information as data linked to an individual or their commonly used device within specific contexts. This draft legislation aims to enhance consumer control over personal information, increase operators’ liability for misuse, and strengthen penalties for violations. Therefore, short video platforms should strictly comply with relevant laws and regulations, handle users’ sensitive personal information in accordance with relevant regulations, and provide prompts to users when obtaining sensitive information, explaining the importance of the information and protecting users’ privacy.

### 6.3 The effectiveness of privacy protection technology and privacy policy negatively affects institutional privacy concerns

Both privacy protection technology and policy effectiveness significantly reduce institutional privacy concerns, indirectly influencing users’ disclosure behaviors through this pathway. However, neither significantly impacts peer privacy concerns. Supporting this, Wang and Herrando (2019) found greater technological efficacy lowers perceived information intrusion. Similarly, Xu et al. (2011) demonstrated effective policies enhance perceived privacy control and reduce perceived risk—aligning with our findings. Collectively, effective technology and policy help regulate user-platform boundary turbulence, facilitate boundary management, and lower perceived privacy risks (Wang et al., 2019). Crucially, peer-related information privacy concerns differ from platform-centric concerns (Zhang et al., 2022). Peer abuse risk depends heavily on social relationships. In high-uncertainty social environments, peer privacy concerns thus center on relationship governance costs (e.g., monitoring fan behavior) rather than platform trust. Privacy technologies and policies primarily govern the user-platform boundary, whereas peer privacy turbulence stems from trust deficits within social relationships. This necessitates relational governance mechanisms—such as layered visibility controls—rather than direct technological or policy solutions.

However, users do not understand the privacy protection technology and privacy protection policy of the platform in the process of using short videos, and may even question it. Therefore, the platform should continuously improve the effectiveness of privacy protection technology and privacy policy. Platforms should continuously upgrade their privacy protection technology to reduce the probability of user information leakage and build the first line of defense with effective privacy protection technology. At the same time, platforms often claim that they use advanced technology to protect users’ privacy, but users do not know which technology is used and how that technology works. Therefore, platforms can use plain and simple language to explain the mechanics of privacy-protecting technologies. In addition, the responsibilities of both parties in ensuring that personal data is protected should be clearly set out in a privacy policy. While privacy policies are obviously placed on the platform’s homepage, privacy policies in short videos are usually presented in the form of links and contain specialized legal terms. Users can simply click “I agree to the terms and conditions” and skip reading the privacy policy. In this regard, platforms should use various forms of digital media (e.g., using images, videos, and animations) to attract users’ attention, promote them to read the full privacy policy (Wang et al., 2019), and popularize the content of the privacy policy.

### 6.4 Peer privacy concerns and institutional privacy concerns negatively influence user privacy disclosure behavior

Both peer and institutional privacy concerns negatively affect users’ privacy disclosure behavior on short-form video platforms. Our finding that heightened institutional privacy concerns reduce disclosure aligns with prior research: users with strong institutional concerns typically disclose less personal information and do so less actively, engagedly, and promptly (Zhang et al., 2022). Similarly, the impact of peer privacy concerns on disclosure behavior is consistent with earlier peer concern studies (Ozdemir et al., 2017). However, this contrasts with Govani and Pashley's (2007) finding that privacy concerns do not significantly affect disclosure—a result often attributed to the privacy paradox. This discrepancy may stem from sample characteristics (student cohorts exhibiting higher risk tolerance) or the amplification of privacy concerns by the strong social attributes inherent to short-video platforms.

Peer and institutional privacy concerns hinge on users’ awareness of privacy issues, highlighting the necessity for improved privacy literacy. Privacy literacy development is facilitated not only by educational institutions and governments but also by short video platforms. These platforms should provide engaging content that enhances users’ understanding of privacy risks and motivates them to protect their data. Moreover, research indicates that smart, personalized advertisements may intensify privacy concerns, potentially causing users to shun recommended ads on social media (Jung, 2017). To mitigate this, short video platforms should employ big data analytics judiciously to obscure user profiles and restrict access to sensitive information, ensuring users’ data is used responsibly. Additionally, analyzing data such as user interactions and video content can help platforms discern users’ preferences for privacy and their tendencies to limit data sharing. Consequently, platforms must embed privacy protection measures throughout all phases of research, development, design, and operations, fostering a secure environment that encourages continued engagement and content creation.

### 6.5 Institutional privacy concerns positively influence peer privacy concerns

The institutional privacy concerns had a positive and significant effect on peer privacy concerns. This is consistent with the findings of Neves et al. (2022) and Liu et al. (2022): when users perceive a high likelihood of institutional data leakage, it also affects their judgment of peer privacy concerns. This discovery reveals a dynamic link between dual privacy concerns in short video contexts: users’ negative assessments of platform data management (institutional privacy concerns) generalize to reevaluate peer behavioral risks, amplifying peer privacy concerns. These results strongly support the boundary synergy perspective in CPM (Petronio, 2002), indicating that institutional and peer privacy boundaries are interdependent and interact via platform-facilitated systemic trust linkages. Moreover, lower institutional privacy concerns—reflecting greater trust in platforms’ protective capacity—reduce users’ defensive vigilance against technological power and mitigate peer interaction risk expectations, ultimately enhancing information disclosure intentions.

Thus, platforms must disrupt the dual concern transmission chain through integrated technology and policy design. Proactive intervention technologies can target peer abuse risks. Forwarding watermarking (e.g., dynamic ID watermarks, non-removable on-screen floats) embeds tracking identifiers in user content, enhancing perceived controllability over dissemination (alleviating peer concerns) while demonstrating platform capabilities to prevent data leaks through technical visibility (mitigating institutional concerns). For real-time sensitive information masking, AI can identify critical elements (e.g., documents, house numbers) and trigger automatic blurring/coding, reducing potential peer misuse at the source. Concurrently, privacy policies must clearly delineate boundaries between “institutional data management” and “peer interaction rules.” Short-video platforms could implement a “graded visibility policy” with refined fan data usage terms (e.g., “fan-visible content prohibits downloading/forwarding,” “intimate circle data excluded from algorithmic recommendations”). This rule transparency reduces users’ anticipated risk of peer boundary violations (curbing peer concerns).

## 7 Conclusion

### 7.1 Theoretical implications

With above findings, this study has significant theoretical implications for research on user privacy disclosure behavior and privacy concern. Firstly, this study enriches the privacy concern literature by including the two facets of privacy concerns (i.e., institutional and peer privacy concerns) into the research on disclosure behavior in information systems. Despite the considerable academic attention received by Internet privacy concerns since their emergence, the majority of studies have primarily centered on controlling personal information disclosure and online engagements with service providers (Dinev and Hart, 2006; Dinev et al., 2013; De Wolf et al., 2014; Xu et al., 2011), neglecting the exploration of peer privacy concerns. Indeed, following a thorough literature review, Zhang et al. (2022) identified this as a prevalent trend in Internet privacy research and advocated for further investigation into peer privacy concerns. In response to their researches, this study not only empirically examines the antecedents of users’ privacy concerns but also establishes users’ institutional and peer privacy concerns as mediators influencing their privacy disclosure behavior. Thus, we contend that this study contributes significantly to a more holistic understanding of user behavior on short-form video platforms. Such contexts are abundant owing to the proliferation of social interaction elements, particularly contemporary technological advancements. Considering that individuals frequently need to share personal information with others (e.g., on online dating platforms, social media, and multiplayer gaming environments), social factors and interactions can potentially evoke peer privacy concerns. Our research underscores the significance of taking into account user privacy risks stemming from peers, as opposed to solely concentrating on threats from website operators.

Second, an evident progression of this study would be to examine privacy concerns as a mediating factor within a broader nomological framework, wherein privacy concerns catalyze behaviors aimed at minimizing privacy disclosure, a process potentially influenced by institutional and peer-related factors. Indeed, several scholars have already explored aspects of this extended model (Bansal et al., 2015; Jozani et al., 2020; Neves et al., 2022; Ozdemir et al., 2017; Wang et al., 2019; Xu et al., 2011). Nevertheless, we posit that a more expansive and holistic model, encompassing not just the precursors and outcomes of privacy concerns, but also explores the dependencies arising from them, would constitute a significant contribution to scholarly literature. Our preliminary findings suggest that both institutional practices and individuals’ perceptions of their peers’ privacy norms induce individuals’ privacy concerns, which in turn reduce users’ privacy disclosure behavior. However, in platforms with social attributes, there may be an interaction between individuals’ perceptions of institutions and individuals’ perceptions of their peers’ behaviors that is complex beyond the threshold of existing theories, and future research is necessary to delve deeper into the interconnections and synergies between these two aspects.

### 7.2 Practical implications

For internet users, privacy may seem psychologically distant and could potentially negatively impact their decisions (Bandara and Perera, 2020). While previous research has primarily focused on the influence of institutional privacy on disclosure behaviors, the threat of peer privacy, which could potentially cause more concern for users (Ozdemir et al., 2017), needs to be considered in the context of users’ self-disclosure behaviors. Our study highlights the significance of institutional and peer privacy concerns in deterring users’ privacy disclosure tendencies on short video platforms, thereby urging platform managers to prioritize the administration of user data and address both institutional and peer privacy concerns. More specifically, it is advocated that managers must safeguard users’ privacy not merely from the short video platforms and associated third-party parties, but also from users’ peers, encompassing individual creators, fans, and visitors. To relieve peer privacy concerns, managers should prioritize enhancing their platforms’ data management systems, stringently restricting access to users’ personal information. Concrete actions could involve refraining from sharing users’ identification data with advertisers, fully empowering users to determine the visibility of their profiles, and anonymizing user reviews, among other measures.

Based on the research findings, this study proposes the following practical countermeasures: (1) Regulatory authorities should strengthen privacy regulations and oversight mechanisms by refining compliance boundaries for data collection, storage, and use in video privacy policies; establishing third-party audits of platform privacy technologies with penalties (e.g., fines, blacklisting); and collaborating with platforms to develop micro-courses enhancing users’ privacy risk identification. (2) Short-video platforms must optimize privacy safeguards through real-time blurring of sensitive video content, restructuring policies with transparent clause summaries, and enhancing social functions allowing follower content restrictions like download prohibitions and forwarding authorizations. (3) Users should proactively manage privacy boundaries by reviewing settings regularly, applying platform blurring tools to highly sensitive content (e.g., family scenes, IDs), and immediately reporting suspected data violations.

### 7.3 Limitations and future research

The conclusions of this study are mainly applicable to algorithm-driven short-video platforms represented by Douyin/TikTok, which indicates that the research has certain limitations. Primarily, it focused exclusively on TikTok users and did not consider those on other short video platforms, where findings might not be universally applicable. Future research should verify the applicability of the model to live-streaming e-commerce platforms (e.g., Taobao Short Videos) and vertical community platforms (e.g., Bilibili), so as to assess the generalizability of the research results across different contexts and user groups.

Additionally, our study did not account for variations in users’ duration of platform use. Longstanding users might exhibit different privacy concerns than newcomers, potentially influencing their privacy disclosure behaviors. Future research should investigate how the duration and frequency of platform use affect privacy concerns and disclosure behaviors, thereby offering a more nuanced understanding of user dynamics over time.

## Data Availability

The data that support the findings of this study are openly available in Science Data Bank at https://doi.org/10.57760/sciencedb.13753.
